# Advances in calcium-sensing receptor modulation: biased signaling and therapeutic potential

**DOI:** 10.1038/s41392-024-02084-9

**Published:** 2024-12-16

**Authors:** Luisa Uhlmann, Ulf Wagner

**Affiliations:** University Leipzig Medical Center, Department for Rheumatology, Leipzig, Germany

**Keywords:** Target identification, Molecular engineering

In a recent study published in *Science*, Liu and colleagues used computational ultra-large library docking to discover new chemotypes acting as positive allosteric modulators (PAM) of the Calcium-Sensing Receptor (CaSR),^1^ which bind the receptor with very high affinity and shows evidence of biased signaling, i.e. the preferential activation of one signaling pathway over others, compared to the receptor’s natural ligands. Importantly, the in vivo test of one substance (‘54159) did not induce hypocalcemia, which is an advantage over FDA-approved calcimimetics, which are limited in clinical practice due to their potential to disrupt calcium homeostasis and cause hypocalcemia.

The Calcium-Sensing Receptor (CaSR) is a G protein-coupled receptor highly expressed in the calcitropic tissues of the parathyroid glands and kidneys. Its central role for calcium homeostasis is based on two main effector mechanisms: activation of parathyroid hormone (PTH) secretion and stimulation of calcium reabsorption in the kidneys. In conditions of insufficient CaSR signaling, calcimimetics are used, which have been developed for the treatment of genetic diseases like familial hypocalciuric hypercalcemia, and autosomal dominant hypocalcemia, but also for the more frequent secondary hyperparathyroidism.

The authors showed, that docking screening with ultralarge libraries generated significantly more hits, characterized by higher potency, while being structurally dissimilar to known PAMs, confirming better performance than docking screening with smaller libraries. Structure-based optimization plays a critical role in refining those initial hits, by modifications altering properties like hydrophobicity or polarity, and therefore enhancing their affinity and potency.

**Fig. 1:**
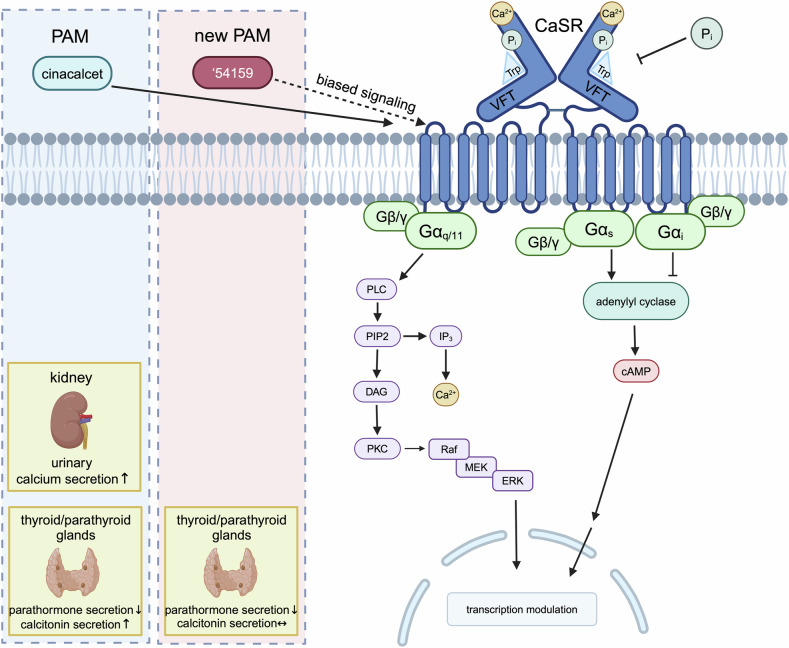
Overview of the calcium sensing receptor (CaSR) signaling in the regulation of parathyroid hormone (PTH) and calcitonin secretion via different G proteins. Beside calcium, various ligands show intrinsic activity on the CaSR. Depending on cell type and modulating factors such as phosphate concentration and allosteric modulators distinct signaling cascades can be triggered influencing the gene transcription. The positive allosteric modulator (PAM) cinacalcet is known to reduce PTH secretion while increasing calcitonin levels, potentially leading to hypocalcemia (dotted blue box). In contrast, the novel PAM suppresses PTH without elevating calcitonin, thereby avoiding hypocalcemia (dotted red box), which might be due to biased signalling or differences in signal transduction in different cell types. CaSR calcium sensing receptor, VFTs Venus flytrap domains, Gα_q/11_/Gα_i_/Gα_s_/Gβγ, different subunits of G proteins, PLC phospholipase C, PIP2 phosphatidylinositol 4,5-bisphosphate, IP3 inositol 1,4,5-triphosphate, DAG diphosphoglycerate, PKC protein kinase C, Raf rapidly accelerated fibrosarcoma, MEK mitogen-activated protein kinase, ERK extracellular signal-regulated kinase, cAMP cyclic adenosine monophosphate, PAM positive allosteric modulator, PTH parathyroid hormone. Figure created with Biorender.com

Structural analysis of the receptor in complex with the newly discovered PAM showed the Venus flytrap domains (VFTs) with bound Ca^2+^, phosphate, and tryptophan in a configuration comparable to the activation of other family C GPCRs. The new PAM induces a distinct active-state CaSR dimer conformation, leading to a more potent suppression of PTH secretion than the approved PAM drugs, while also requiring lower doses, in vivo. This might be due to the promotion of a conformation closer to the G protein-coupled state than other drugs. Importantly, it induced less hypocalcemia than cinacalcet, which might be attributed to biased signaling of the CaSR resulting in less CaSR-mediated calcitonin secretion. For some PAMs other than cinacalcet, biased signaling towards the ERK1/2 pathway has been described to be the reason for PTH suppression in parathyroid cells without induction of calcitonin secretion in parafollicular cells of the thyroid gland. However, the specific signaling pathways activated by this new PAM remain to be fully elucidated (Fig. 1).

Besides calcium, CaSR is also sensing extracellular amino acid and phosphate concentrations thereby acting as a phosphate receptor. Moreover, it continues to signal after endocytosis from the endosomal pathway. Binding of phosphate ions to the CaSR has the consequence of reduced ligand induced-signaling, which is of relevance in patients treated with calcimimetics, because it was found that calcimimetic-induced decreases in serum PTH concentrations were reduced in individuals with higher serum PO4 levels.^2^ This inhibitory influence of high phosphate concentrations, which are often present in patients with hyperparathyroidism, might be overcome by the newly developed chemical PAM due to its higher affinity to the receptor.

More recently, expression of CaSR in non-calcitropic tissues has gained attention, where it’s dysfunction or dysregulation has been associated with Alzheimer’s disease, epilepsy, ischemic brain injury, but also with cardiovascular complications like myocardial ischemia, vascular calcifications, hypertension, obesity and arteriosclerosis. In innate immunity, the receptor plays a pivotal role in the initiation and regulation of constitutional macropinocytosis of myeloid cells and is required for calcium induced NLRP3 inflammasome activation in monocyte and macrophages.^3^

In many conditions, however, the precise mechanism of CaSR involvement is not clear. Whenever the receptor appears to play an anti-inflammatory role, treatment with calcimimetics might be beneficial.

In the lower gastrointestinal tract, for example, CaSR is expressed in most sectors of the intestine and regulates fluid absorption, intestinal motility, and the secretion of digestive hormones and electrolytes. Several studies have shown that the calcium sensing receptor plays a protective role in intestinal inflammation. CaSR signaling is also essential for maintaining intestinal barrier integrity and regulation of inflammation. Loss of CaSR function in the intestinal epithelium has been associated with altered gut microbiota composition, modified expression of intestinal pattern recognition receptors, and increased susceptibility to inflammatory bowel diseases.^4^

Accordingly, calcimimetics have been suggested and pre-clinically tested for treatment of chronic inflammatory bowel diseases. There are, however, also conflicting data describing a more proinflammatory role for the receptor in the intestine, and new data are clearly required before clinical trials with newly developed calcimimetics could be started. Importantly, intestinal CaSR may mediate the gastrointestinal side effects seen in patients on long-term calcimimetic treatment, and the precise effects of new, highly potent calcimimetics with biased signaling different from the FDA approved substances need to be evaluated.

In the cardiovascular system, CaSR activation can improve cardiac remodeling in hypertensive models, indicating a protective role of CaSR against hypertrophy.^5^ This protective mechanism is crucial, as cardiac hypertrophy is a significant risk factor for heart failure and ischemic heart disease. It has also been shown in vitro, that ischemia increases CaSR expression in cardiomyocytes and leads to apoptosis, while activation of the CaSR mediates the cardioprotection, which can be induced by ischemic preconditioning of cardiomyocytes. And finally, in calciphylaxis, calcimimetics are used to improve not only calcium deposition in the skin, but also vascular calcifications, and it is tempting to speculate whether this therapeutic principle might also hold promise in other forms of accelerated arteriosclerosis. Accordingly, preclinical and clinical studies of potentially beneficial effects of PAMs of the CaSR in the cardiovascular system are warranted.

Calcimimetics have also been shown in pre-clinical models to improve bone regeneration and de novo bone formation, and to increase the efficiency of mesenchymal stem cells developed for therapeutic applications in bone damage. New PAMs might even revitalize studies investigating the use of calcimimetics in osteoporosis.

Several pathological conditions, such as allergic asthma and rheumatoid arthritis, have been associated with a pro-inflammatory and disease-aggravating role for CaSR, often combined with increased expression of the receptor.^3^ The precise molecular pathways are not entirely clear in those conditions, and it is not known in detail, whether upregulated expression is always accompanied by increased signaling from the receptor, or whether dysregulation might play a role. Therefore, high affinity PAMs of CaSR with biased signaling might also be beneficial and might be worthwhile to explore.
